# Supplementary material for: Reliability of radiologists’ first impression when interpreting a screening mammogram
Source: PLoS One. 2023 Apr 25;18(4):e0284605. doi: 10.1371/journal.pone.0284605 (PMC10128970; doi:10.1371/journal.pone.0284605)
Supplement: S1 File — (DOCX) [file pone.0284605.s001.docx]

**Supplementary Analysis 1: Range of gist scores given to the images**

To ensure that the included set of images resulted in low-, intermediate-, and high-gist scores, the range of the provided gist scores were explored. In total 12480 (39 Readers × 2 sessions each × 160 images) ratings were available. The histogram showing the distribution of these scores is shown in Supp Fig 1. As indicated, the provided scores cover a wide range.

**Supp Fig. 1. The distribution of gist scores. Scores from all trials (72 sessions, each including 160 images) were included.**

**Supplementary Analysis 2: The association between observers’ performances in two rounds**

The AUC values of all 39 radiologists in two rounds are indicated for each category in Supp Fig 2. In addition to the analysis presented in Section 3.1, we explored the correlation among the AUC values across the classes to investigate whether a reader’s performance in detecting cancer on a cancer-containing image is associated with their performances in predicting future cancer. The correlation coefficients are shown in Supp Fig. 3. In addition to the AUC values for each classification task in each round, we calculated an average gist score per case, by averaging the abnormality scores given to each case in two rounds by a reader. By doing so, intra-observer noise was canceled out to some extent. The results based on these average scores are shown by the “All” index in Supp Fig. 3. As shown, performances demonstrated generally good consistency across categories when the scores from two rounds were averaged for each case, as shown by the “All” results in the figure compared to the 1 or 2 results.


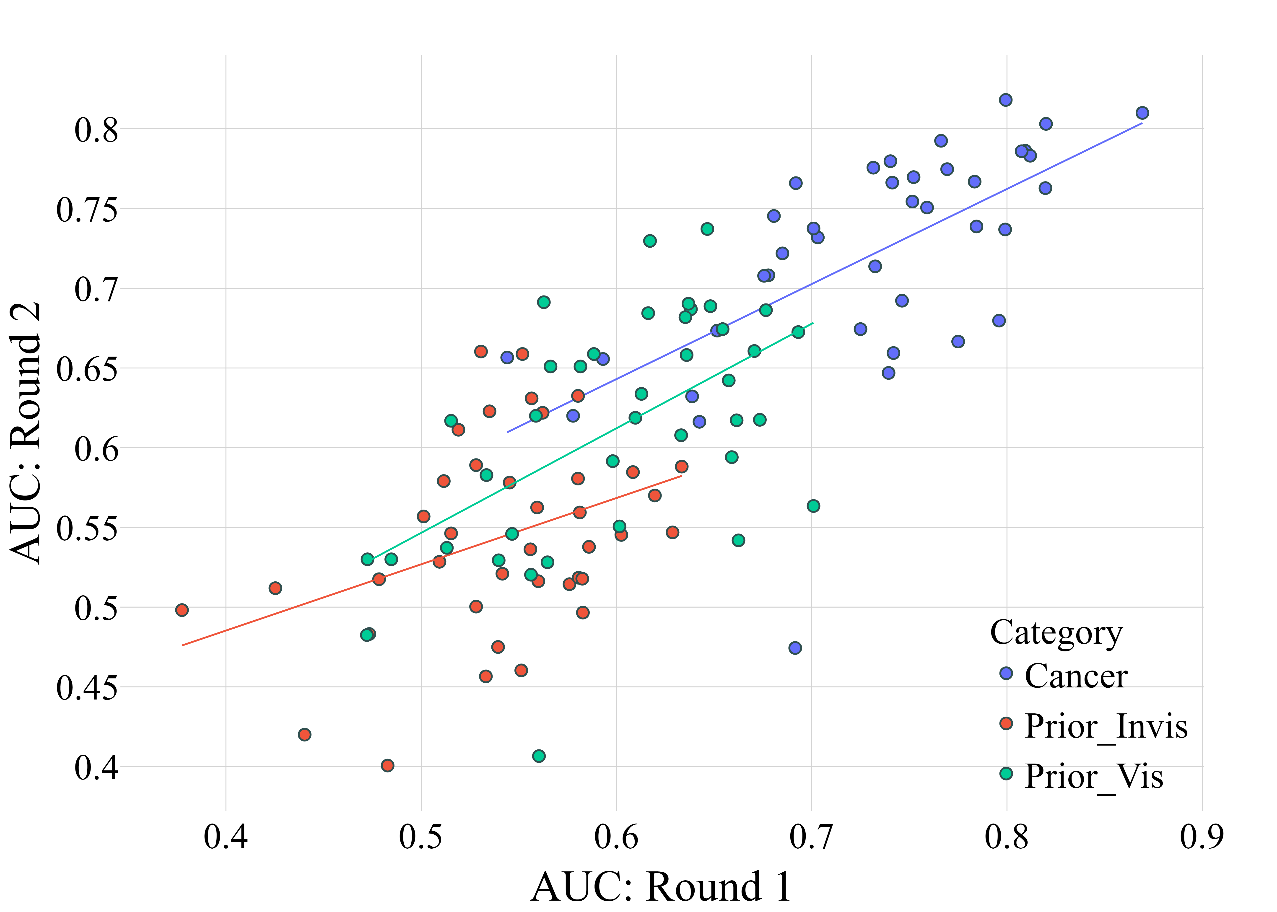


**Supp Fig. 2. The area under the receiver operating characteristics curve (AUC) for each category in the first (AUC: Round 1) and second (AUC: Round 2) rounds.**

**
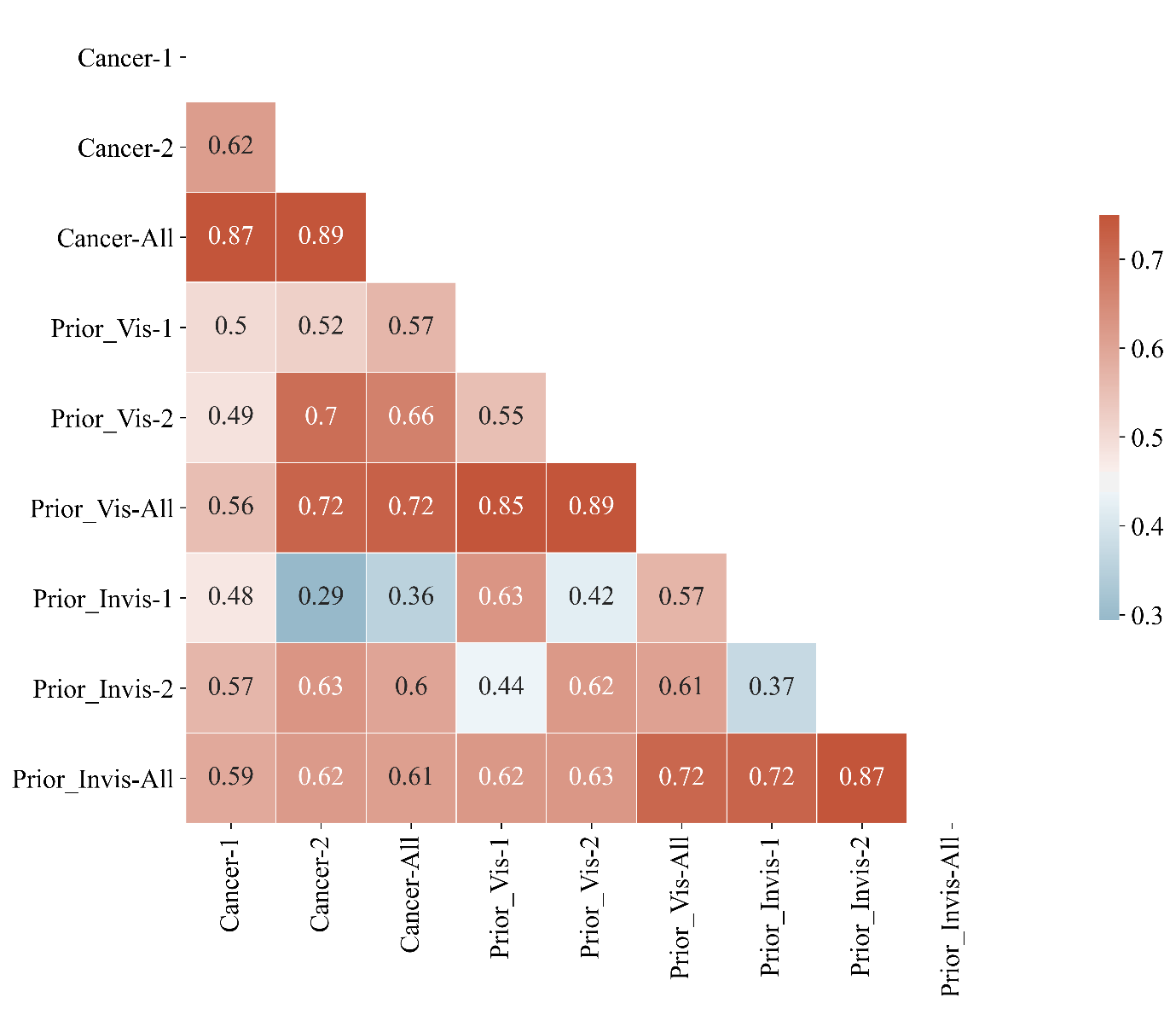
**

**Supp Fig. 3. The Pearson’s correlation coefficient between the AUC values for all possible pairs. In each category three values, representing the first round (1), the second round (2), and the average of two round (All) is shown. For example, the highlighted item in the last row shows the correlation between AUC values for discriminating Prior_Invis images from Normal ones based on the average of gist scores from two rounds and the AUC values for discriminating Cancer images from Normal ones in the first round**.

**Supplementary Analysis 3: Image category and reader characteristics as predictors of the gist scores**

The gist of the abnormal can indicate the presence of current breast cancer as well as an elevated risk of future breast cancer [8]. In cancer-containing cases, both “what” and “where” information could lead to the gist response. In high-risk cases, which do not contain an overt cancer sign, the “what” signal drives the gist of the abnormal, perceived by the observers. Such a signal could be related to the overall mammographic texture. This is coherent with the findings from an extensive body of literature, which shows the computer-extracted textural features extracted from the entire breast tissue on mammograms, can predict a future breast cancer at above chance-level. Our earlier studies showed that when the gist signal solely relies on “what” information, it is less strong. It is unknown if only a subset of readers has the capability of perceiving gist information related to predicting a future breast cancer.

We constructed a linear mixed model to investigate if all readers have the capability of perceiving gist information related to predicting a future breast cancer or only “Gist Experts” has such capability. In the model, the “image category” was treated as a fixed effect and a predictor of the gist scores. Reader and image were considered as random effects. Treating both reader and case as random effects is a popular approach in multi-reader multi-case studies [23-25]. Using interaction terms between the image category and the reader characteristic, we tested the hypothesis that only certain readers (e.g., “Gist Experts” or readers with outlier Reader Characteristics) would be better at identifying the gist of the abnormal in specific image categories. As an example, the interaction term “Cancer **|** Gist Expert”, estimates if on average the “Gist Experts” assigned additional points to a Cancer case, compared to the estimates for the term “Cancer: Normal” in our experiment. To avoid bias, only the readings from the first round were used to categorize readers as “Gist Experts” and the model was fitted to the gist scores from the second round. In addition to a flag showing if the reader is a Gist Expert, three significant variables from Tables 2 and 3 (please refer to the paper) were also fed into the model. The coefficients, their confidence intervals, and the corresponding p-values for each variable are shown in Supp Table 1.

As shown in Supp Table 1, the average gist score of a Cancer case was 14.73 [CI: 7.25-22.21] rating points greater than that of a “Normal” case. Moreover, “Gist Experts”, assigned an additional 3.42 [CI: 0.5-6.33] points to a “Cancer” case rating. Readers’ workload level (number of screening mammograms per week) was discretized into four levels, representing four quartiles. On average, each increased level of workload added 1.66 [CI: 0.36-2.97] points to the rating on an average Cancer case. Similarly, the number of breast biopsy examinations in the last 12 months was discretized into four levels. For each increased level of this variable, the model indicated an increase of 1.58 [CI: 0.2-2.95] points to the Cancer rating. “Gist Experts” also assigned a significantly higher abnormality rating to cases in “Prior_Vis” (5.72 [CI: 2.81-8.64] added points) and “Prior_Invis” (5.04 [CI:2.12-7.95] added points) categories.

**Supp Table 1- The linear mixed model for predicting the gist scores with case id and reader id as the random effects.**

| **Name** | **Estimate** | **p-value** |
| --- | --- | --- |
| Intercept | 27.93 [22.85-33.01] | **<0.00001** |
| Cancer:Normal^*^ | 14.73 [7.25-22.21] | **0.0001** |
| Prior_Vis:Normal^*^ | 5.42 [-2.06-12.91] | 0.1552 |
| Prior_Invis:Normal^*^ | 1.12 [-6.36-8.6] | 0.7694 |
| Cancer\|Gist Expert^#^ | 3.42 [0.5-6.33] | **0.0215** |
| Prior_Vis\|Gist Expert^#^ | 5.72 [2.81-8.64] | **0.00011** |
| Prior_Invis\|Gist Expert^#^ | 5.04 [2.12-7.95] | **0.00070** |
| Cancer\|weekly casesˆ | 1.66 [0.36-2.97] | **0.01239** |
| Prior_Vis\|weekly casesˆ | 0.18 [-1.12-1.48] | 0.78805 |
| Prior_Invis\|weekly casesˆ | -0.02 [-1.32-1.29] | 0.98022 |
| Cancer\|#biopsies^⁋^ | 1.58 [0.2-2.95] | **0.02485** |
| Prior_Vis\|#biopsies^⁋^ | 1.02 [-0.36-2.39] | 0.14853 |
| Prior_Invis\|#biopsies^⁋^ | -0.04 [-1.42-1.34] | 0.95268 |
| Cancer\|Diagnostic focus^†^ | -0.89 [-2.31-0.53] | 0.22032 |
| Prior_Vis\|Diagnostic focus^†^ | -0.7 [-2.12-0.72] | 0.33235 |
| Prior_Invis\|Diagnostic focus^†^ | 0.32 [-1.1-1.74] | 0.66175 |

*^*^ Category effects are measured relative to Normal*

*^#^ An interaction term, showing the interaction between the category and the Gist Expert flag (0 or 1) based on the first round.*

*ˆ An interaction term, showing the interaction between the category and the weekly number of cases, the variable is discretised into four quartiles.*

*^⁋^ An interaction term, showing the interaction between the category and number of biopsy examinations in the last 12 months, the variable is discretised into four quartiles.*

*^†^An interaction term, showing the interaction between the category and the percentage of time dedicated to reading diagnostic mammograms, the variable is discretised into four quartiles.*

**Supplementary Analysis 4: The intra-reader reliability of the gist signal**

To explore the intra-reader reliability of the gist signal, the intraclass correlation coefficient (ICC) and weighted Cohen’s kappa were used. Supp Table 2 shows the values of ICC and weighted Kappa for all readers and Supp Fig 4 illustrates the distribution of these two metrics for “Gist Experts” and “Others”.

**Supp Table 2- The intraclass correlation coefficient (ICC) and weighted Cohen’s kappa for each radiologist is shown. The values show the intra-reader reliability of the signal. The p-values show if the agreement level is significantly different form the chance level.**

| Reader# | ICC [95% Confidence Interval], p-value | Weighted Kappa [95% Confidence Interval], p-value |
| --- | --- | --- |
| 1 | 0.47 [0.34-0.58], p<0.0001 | 0.47 [0.27,0.67], p<0.0001 |
| 2 | 0.47 [0.34, 0.58], p<0.0001 | 0.43 [0.25,0.61], p<0.0001 |
| 3 | 0.62 [0.52, 0.71], p<0.0001 | 0.53 [0.32,0.74], p<0.0001 |
| 4 | 0.63 [0.52, 0.71], p<0.0001 | 0.6 [0.43,0.78], p<0.0001 |
| 5 | 0.55 [0.44, 0.65], p<0.0001 | 0.7 [0.54,0.86], p<0.0001 |
| 6 | 0.61 [0.5, 0.7], p<0.0001 | 0.4 [0.21,0.58], p<0.0001 |
| 7 | 0.46 [0.33, 0.58], p<0.0001 | 0.61 [0.43,0.78], p<0.0001 |
| 8 | 0.43 [0.3, 0.55], p<0.0001 | 0.61 [0.43,0.78], p<0.0001 |
| 9 | 0.53 [0.4, 0.63], p<0.0001 | 0.51 [0.32,0.7], p<0.0001 |
| 10 | 0.61 [0.51, 0.7], p<0.0001 | 0.44 [0.24,0.64], p<0.0001 |
| 11 | 0.38 [0.24, 0.5], p<0.0001 | 0.29 [0.05,0.54], p=0.0005 |
| 12 | 0.49 [0.37, 0.6], p<0.0001 | 0.69 [0.54,0.85], p<0.0001 |
| 13 | 0.41 [0.27, 0.53], p<0.0001 | 0.5 [0.31,0.68], p<0.0001 |
| 14 | 0.43 [0.29, 0.55], p<0.0001 | 0.48 [0.3,0.66], p<0.0001 |
| 15 | 0.51 [0.38, 0.61], p<0.0001 | 0.38 [0.16,0.61], p<0.0001 |
| 16 | 0.52 [0.4, 0.63], p<0.0001 | 0.41 [0.2,0.62], p<0.0001 |
| 17 | 0.71 [0.63, 0.78], p<0.0001 | 0.48 [0.28,0.68], p<0.0001 |
| 18 | 0.41 [0.28, 0.53], p<0.0001 | 0.5 [0.3,0.7], p<0.0001 |
| 19 | 0.35 [0.21, 0.48], p<0.0001 | 0.52 [0.26,0.78], p<0.0001 |
| 20 | 0.36 [0.22, 0.49], p<0.0001 | 0.47 [0.26,0.67], p<0.0001 |
| 21 | 0.5 [0.37, 0.61], p<0.0001 | 0.42 [0.2,0.63], p<0.0001 |
| 22 | 0.72 [0.64, 0.79], p<0.0001 | 0.68 [0.51,0.85], p<0.0001 |
| 23 | 0.51 [0.38, 0.61], p<0.0001 | 0.55 [0.38,0.73], p<0.0001 |
| 24 | 0.5 [0.38, 0.61], p<0.0001 | 0.42 [0.22,0.62], p<0.0001 |
| 25 | 0.59 [0.47, 0.68], p<0.0001 | 0.4 [0.19,0.61], p<0.0001 |
| 26 | 0.34 [0.2, 0.47], p<0.0001 | 0.54 [0.3,0.77], p<0.0001 |
| 27 | 0.39 [0.25, 0.51], p<0.0001 | 0.49 [0.29,0.68], p<0.0001 |
| 28 | 0.54 [0.42, 0.64], p<0.0001 | 0.33 [0.12,0.55], p<0.0001 |
| 29 | 0.69 [0.6, 0.76], p<0.0001 | 0.47 [0.27,0.68], p<0.0001 |
| 30 | 0.51 [0.39, 0.62], p<0.0001 | 0.66 [0.5,0.82], p<0.0001 |
| 31 | 0.76 [0.68, 0.82], p<0.0001 | 0.71 [0.55,0.86], p<0.0001 |
| 32 | 0.59 [0.47, 0.68], p<0.0001 | 0.57 [0.35,0.79], p<0.0001 |
| 33 | 0.66 [0.56, 0.74], p<0.0001 | 0.45 [0.26,0.64], p<0.0001 |
| 34 | 0.53 [0.41, 0.64], p<0.0001 | 0.47 [0.26,0.67], p<0.0001 |
| 35 | 0.47 [0.34, 0.58], p<0.0001 | 0.53 [0.35,0.72], p<0.0001 |
| 36 | 0.69 [0.59, 0.76], p<0.0001 | 0.34 [0.19,0.48], p<0.0001 |
| 37 | 0.62 [0.52, 0.71], p<0.0001 | 0.32 [0.07,0.57], p<0.0001 |
| 38 | 0.68 [0.59, 0.76], p<0.0001 | 0.64 [0.47,0.82], p<0.0001 |
| 39 | 0.63 [0.53, 0.72], p<0.0001 | 0.33 [0.13,0.54], p<0.0001 |


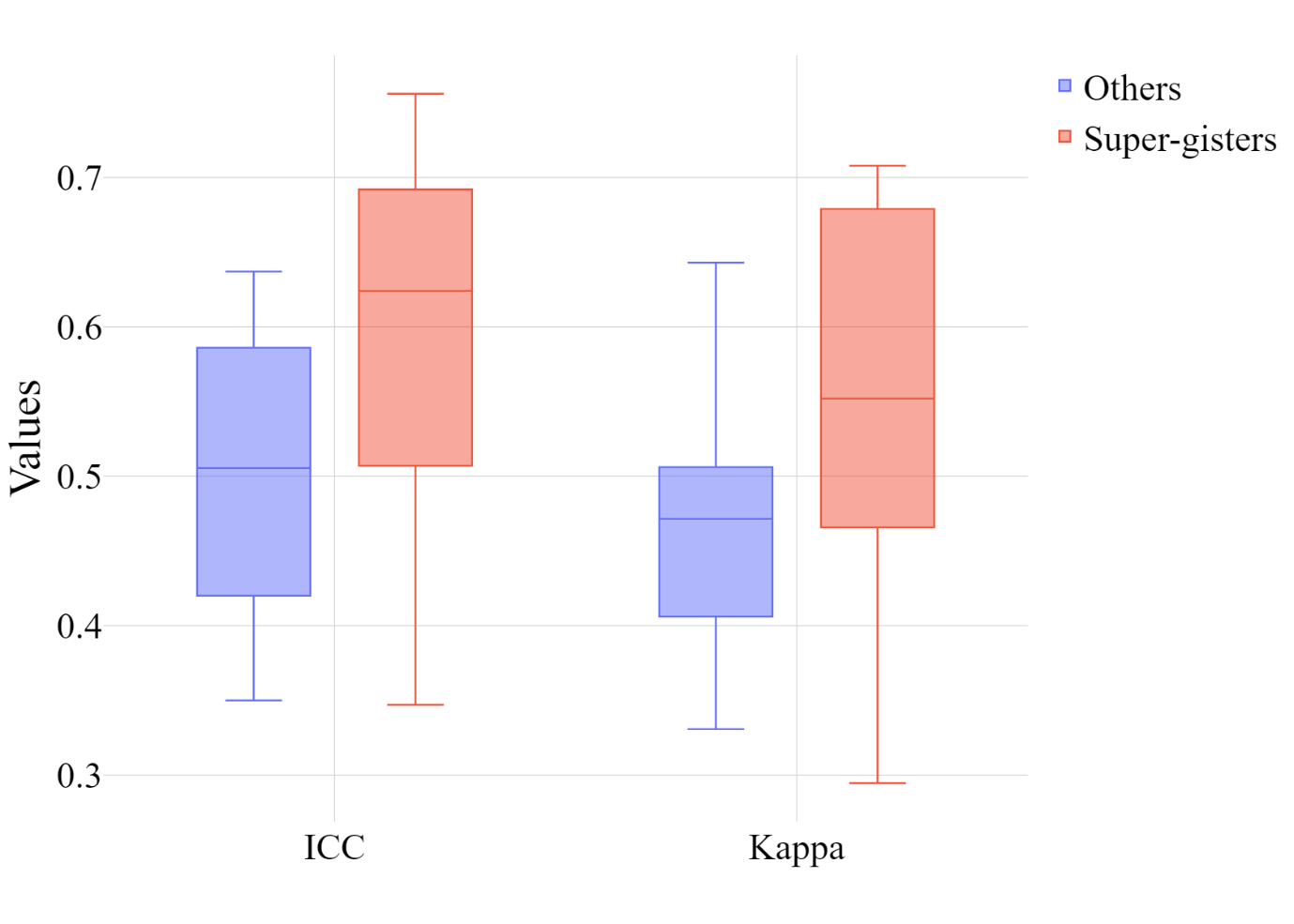


Others

Gist Experts

**Supp Fig. 4. The distribution of intra-class correlation (ICC) and Weighted Cohen’s Kappa (Kappa) for measuring the agreement between the readers’ gist scores in two rounds. The data for “Gist Experts” and “others” were shown separately. The median intra-reader ICC for the “Gist Experts” was 0.63 (IQR: 0.51-0.691) while the median intra-reader ICC for the other group was 0.51 (IQR: 0.42-0.59). The intra-reader Cohen’s kappa was on average 0.50±0.11 (moderate agreement) and ranged from 0.29 to 0.71 for 39 readers while on average the inter-reader Cohen’s kappa was 0.09±0.05 (slight agreement).**
