# Supplementary material for: Reliability of radiologists’ first impression when interpreting a screening mammogram
Source: PLoS One. 2023 Apr 25;18(4):e0284605. doi: 10.1371/journal.pone.0284605 (PMC10128970; doi:10.1371/journal.pone.0284605)
Supplement: Attachment — Submitted filename: Responses.docx [file pone.0284605.s002.docx]

We would like to thank the editor and the reviewers for their valuable comments, which we believe have greatly improved the manuscript. Responses to specific comments made can be found below. The changes are also highlighted in the manuscript.

Also, all de-identified collected data from radiologists, used in this study will be available through the following Kaggle dataset link:

<https://www.kaggle.com/datasets/zibaga/gist-of-the-abnormal-on-screening-mammograms>

PCB, JMW, SJL, KKE, DX, and ZG was funded by National Health and Medical Research Council (grant number APP1162872). Also, PCB, JMW, SJL, and KKE was funded by National Breast Cancer Foundation (IIRS-18-089). The funders had no role in study design, data collection and analysis, decision to publish, or preparation of the manuscript. We can confirm that we understand the above-mentioned statements will be included as “Funding Statement” in the manuscript.

The authors have declared that no competing interests exist.

**Point by point responses to the comments:**

Reviewer #2:

*1) I have some concerns, mainly of clinical character, that could be clarified by some additions. A broaden description of the cases included in the experiments are warranted (Materials and* Methods*/Mammograms).* *Did normal cases include benign findings or no findings at all? If no findings, we cannot exclude that also benign findings evoke a gist signal with the same magnitude as cancer cases. The gist signal might then not be as “useful” in a clinical setting where there are many benign findings. I can understand that you* *pick out the* extremes *(normal-abnormal) to study the gist signal, but this limitation should then be included in the discussion.*

Thank you very much for the comment. To address this comment, we added this point as a limitation of our study and acknowledged that we only focused on two extremes (normal-abnormal) in this paper to study the gist signal.

The abnormal categories were “Cancer”, “Prior_Vis”, and “Prior_Invis”. Certain types of benign findings are associated with an elevated risk of future breast cancer. As stated, two types of gist signal exist, one providing “where” information (to guide the gaze to suspicious areas) and one providing “what” information (to globally assess the image and alert the radiologist that the mammogram represents a high-risk patient). Therefore, the latter type of the gist signal could be present in these benign cases. If the purpose of using the gist signal is the prediction of a future malignancy, the existence of the gist of the abnormal on these benign cases could be desirable. However, further studies are required to compare the strength of the gist signals on mammograms from patients with non-proliferative benign breast diseases and proliferative benign breast diseases. As existing literature suggests that only cases with proliferative benign breast diseases demonstrated an increased risk of a future malignancy [1]. Therefore, in future, it should be explored if the gist signal is stronger in these cases compared to cases with non-proliferative benign breast diseases.

**References**

1.	Dyrstad SW, Yan Y, Fowler AM, Colditz GA. Breast cancer risk associated with benign breast disease: systematic review and meta-analysis. Breast Cancer Res Treat. 2015;149(3):569-75. Epub 2015/02/01. doi: 10.1007/s10549-014-3254-6. PubMed PMID: 25636589.

*2) You state that all cancer cases were subtle. What is your definition of a subtle cancer case? Do you have some numbers to support the case selection, for example tumor size? The case illustrated in Fig 1. does not look like a typically subtle case.*

Thank you for the comment. We deleted the word “subtle” and included the tumor size of the “Cancer” cases. All cases were retrieved from an archive of BreastScreen Australia, where the standard practice is double reading. The selected “Cancer” cases were missed by one of the two radiologists who assessed the cases. We added this to the manuscript instead of using the word “subtle”.

*3) Since breast cancer is a heterogeneous disease with different mammographic appearances, and therefore more or less difficult to detect, it could also be relevant to include tumor types. I wonder for example if invasive lobular cancer or microcalcification cases were included?*

Thank for the comment. To address the comment, we added a table and provided the characteristics of lesions and also the breast density of the cases. However, the histopathological reports of the cases were not available. We acknowledged this as a limitation of our study in the discussion. This is an interesting future work, as the aggressiveness of various cancer types differs, and this could impact the gist strength.

*4) Concerning the readers. Could you check the validity that the readers on average read 200 cases per week/10,000 per year working 40% with breast imaging? It raises some concerns on the self-reported statistics (I am not implying that the readers are lying, but it can perhaps be hard to estimate your own work).*

Thank you very much for raising this interesting point. We added a few sentences in the discussion and explained that we relied on self-reported metrics and these metrics could be over-estimated. As the reviewer suggested, previous studies also raised concerns about the accuracy of self-reported reader characteristics.

One possible explanation for these numbers is that there is a well-known problem of radiology workforce shortage in Australia and New Zealand [1-3]. Therefore, radiologists might work very long hours on their working days. Actually, on average radiologists in Australia and New Zealand work more than one full-time employee (FTE) [4].

In terms of the possibility of reading 200 cases per week, based on our own experiments at BreastScreen Reader Assessment Strategy (BREAST) workshops, on average, it took two hours for a screen reader to interpret a test of 60 cases. This equates to approximately 7 hours for interpreting 200 cases. Based on another study, on average, it took 240 seconds to interpret a digital mammogram [5]. Considering this figure, reading 200 cases equates to 13 hours. This study was conducted more than a decade ago, so considering the technological advancements, currently, the required time could be even lower. Considering this number, it may take 2 days to read 200 images. With workforce productivity of 75%, this equates to 40% of a working week if the individual works full-time.

In Australia and New Zealand, there are about 2000 radiologists, and based on a self-reported survey about 14% of these radiologists are screen readers (in our cohort only 5 were not screen readers). In total, in each round of screening (i.e., 2 years), about 2.2M screening exams are produced. Because the standard practice is double reading with the arbitration, more than 2.2M assessments are conducted each year. Therefore, the average reading volume of screen readers is more than 7857 (2.2M assessments / (2000*0.14 screen readers). This equates to 164 cases per week (a person has 48 working weeks in a year in Australia and New Zealand). Therefore, even though it is close to 200, as raised by the reviewer, there is a probability that the respondents overestimated their reading volume. This is coherent with concerns raised by earlier studies [6].

**References**

1. Brooks PM, Lapsley HM, Butt DB. Medical workforce issues in Australia:" tomorrow's doctors-too few, too far". Medical Journal of Australia 2003;179:206-208.

2. Patty A. Radiologist shortage for breast scans. Daily Telegraph 2003.

3. Association AM. AMA blames Qld Health for radiologist shortage. ABC Premium News, Sydney 2006.

4. RANZCR, 2016 Australian radiology workforce report. Available from <https://www.ranzcr.com/documents/4624-2016-clinical-radiology-workforce-census-report-australia/file>

5. Haygood TM, Wang J, Atkinson EN, Lane D, Stephens TW, Patel P, Whitman GJ. Timed efficiency of interpretation of digital and film-screen screening mammograms. AJR. American journal of roentgenology. 2009 Jan;192(1):216.

6. Smith-Bindman R, Miglioretti DL, Rosenberg R, Reid RJ, Taplin SH, Geller BM, et al. Physician workload in mammography. American Journal of Roentgenology. 2008;190(2):526-32.

*How come the time dedicated to reading diagnostic mammograms (diagnostic focus) was associated with gist experts in Table 2 when it was an unsignificant factor in Table 3, or do I read it wrong?*

Thank you for the comment. That is an interesting observation. There are a few explanations for this:

(1) Due to the small sample size the correlation coefficients for the diagnostic focus did not yield a significant p-value, although it is above 0.2 and it is still more than most of the other characteristics. Larger sample size could lead to a significant p-value. This is added to the Discussion.

(2) Also, the correlation coefficients were calculated for each pairwise comparison while categorizing readers as “Gist Experts” and “Others” were done based on the average of the AUCs from three pair-wise comparisons. As stated, the gist is a noisy signal, and averaging these three AUC values could reduce the noise and produce a performance metric more reflective of the user’s ability in detecting the gist signal. This explanation is added to the Result section.

(3) for calculating the correlation coefficients, both gist experts and non-experts were pooled together. As an example, the scatter plot presented in Figure 1 shows the association between the AUC values for Cancer vs Normal categorization and the diagnostic focus. The red dots showed the “Gist Experts” while the blue dots showed “Other” radiologists. Within the “Other” group, a non-significant negative correlation (r=-0.21, p=0.30) and within the “Gist Experts”, a non-significant positive correlation (r=0.16, p=0.62) was noted. Figure 2 presents the boxplots for each category. As shown, the “Other” group included several radiologists, who stated that their diagnostic focus is less than 2.5% (about half of them). Even though the data points corresponding to these people reduced the correlation coefficient, the association of the diagnostic factor with being a “Gist Expert” was captured by the non-parametric test, i.e., Mann-Whitney U-test (p=0.004).

Figure 1- A scatter plot showing the AUC values for “Cancer” vs “Normal” categorization against diagnostic focus.

Figure 2- Box plots showing the distribution of the AUC values for “Cancer” vs “Normal” categorization for “Gist Experts” and “Others”.

*A few minor comments:Abstract: The labels C_high_ and C_low_ do not appear later in the text. (Why C? It refers to high and low gist signal, right?)*

Thanks for raising this. Yes, that is correct. We omitted these abbreviations in the abstract. Previously they were only used in the abstract to ensure its word count is the required word limit.

*Materials and Methods: 2-2 Mammograms. Explain CC.*

Thanks for the comments. Fixed.

*Results: Check “Prio_Vis” for consistency (incl. Discussion).*

Thanks for raising this point. We fixed it throughout the manuscript.
