# Supplementary material for: Reliability of radiologists’ first impression when interpreting a screening mammogram
Source: PLoS One. 2023 Apr 25;18(4):e0284605. doi: 10.1371/journal.pone.0284605 (PMC10128970; doi:10.1371/journal.pone.0284605)
Supplement: Attachment — Submitted filename: Responses_1_09_2022.docx [file pone.0284605.s003.docx]

We would like to thank the editor and the reviewer for their valuable comments, which we believe have greatly improved the manuscript. Responses to specific comments made can be found below. The changes are also highlighted in the manuscript.

Also, all de-identified collected data from radiologists, used in this study will be available through the following Kaggle dataset link:

<https://www.kaggle.com/datasets/zibaga/gist-of-the-abnormal-on-screening-mammograms>

PCB, JMW, SJL, KKE, DX, and ZG was funded by National Health and Medical Research Council (grant number APP1162872). Also, PCB, JMW, SJL, and KKE was funded by National Breast Cancer Foundation (IIRS-18-089). The funders had no role in study design, data collection and analysis, decision to publish, or preparation of the manuscript. We can confirm that we understand the above-mentioned statements will be included as “Funding Statement” in the manuscript.

The authors have declared that no competing interests exist.

**Point by point responses to the comments:**

*Reviewer #3:*

*The manuscript is very difficult for reading. I suggest authors to perform substantial rewriting leaving only the most important information as main text. Additional analysis should be presented as supplemented material (for example, Tables 2, 4, Figures 2, 3, 4, 5, 6, 7, 8).
Authors should follow GRRAS guidelines (PMID: 21130355 DOI: 10.1016/j.jclinepi.2010.03.002)*

Thank you very much for the comment. We believe addressing the comment considerably improved the presentation of our manuscript. Briefly, we amended the introduction and stated that the current study has four distinct aims. One of these aims (Aim 2-2) was to explore the test-retest reliability of the gist signal. To realize this aim we ensured that the GRRAS guideline is followed. Table Res. 1 shows the criteria in the GRRAS guidelines and how we addressed the criteria.

While aim (2-2) investigates how reliable the gist signal is, aim (1) explores if the overall performance of a reader across all cases in two rounds (intra-radiologist variability in the performance) was associated. If the gist signal is generally present across the abnormal cases but picked up in a stochastic manner by an observer, the observer could detect the abnormality signal in one round but could miss it in another round. In such a scenario, an observer’s overall performance could be almost similar in two rounds, however, each time they detect the gist of the abnormal in different cases. This translates into low intra-radiologist variability in the overall performance but poor test-retest reliability of the gist signal. As our study showed that the intra-observer reliability is poor to modest, we also explored the magnitude of intra-observer variation in overall performance. Our data suggested that, when the gist signal is strong enough, i.e., with the cancer cases, the performance level in the first round of the gist experiment remained almost consistent in the second round. We also explored if we could predict which radiologists have a superior gist capability based on their characteristics (aim 2-1). Finally, aims (3) and (4) focus on the cases-level analysis to provide recommendations about whether an observer should trust their initial impression when a very strong gist of the abnormal is noted on a mammogram or conversely in the absence of a sense of an abnormal gist. This is important from a clinical point-of-view as it is unknown whether, in these extreme occasions, an observer’s initial impression can be trusted. Our findings suggest that the gist of the abnormal, especially a strong one, should not be ignored, because when the gist is strong, the probability of a case being abnormal is significantly higher than the probability that it is normal. Conversely, in the absence of a sense of an abnormal gist, readers should keep searching because the absence does not reliably signal a normal case.

Moreover, as suggested by the reviewer, some of the analyses, tables, and figures are now moved to Supplementary Materials.

Table Res. 1- The criteria in the GRRAS guidelines (Italic) and how we addressed them.

| Title and abstract | 1. | *Identify in title or abstract that interrater/intrarater reliability or agreement was investigated*  In the abstract, it is stated that the study explored the intra- and inter-observer reliability of the reader’s gist signal. |
| --- | --- | --- |
| Introduction | 2. | *Name and describe the diagnostic or measurement device of interest explicitly*  Stated. |
|  | 3. | *Specify the subject population of interest*  Stated. |
|  | 4. | *Specify the rater population of interest (if applicable)*  Stated. |
|  | 5. | *Describe what is already known about reliability and agreement and provide a rationale for the study (if applicable)*  Stated (The reliability is not known. The current study aimed at addressing this gap in the literature). |
| Methods | 6. | *Explain how the sample size was chosen. State the determined number of raters, subjects/objects, and replicate observations.*  Provided in the Sample size calculation. |
|  | 7. | *Describe the sampling method*  Participants (raters): the details are provided in Section 2-3 (Experimental Design and Study Participants).  Images (objects): the details are provided in Section 2-4 (Mammograms). Further explanation is provided in the Supplementary Analysis 1. |
|  | 8. | *Describe the measurement/rating process (e.g., time interval between repeated measurements,* *availability of clinical information, blinding)*  The details are provided in Section 2-3 (Experimental Design and Study Participants) and Section 3-1 (“On average, readers conducted the second round of the readings 7±1 weeks after the first round”). |
|  | 9. | *State whether measurements/ratings were conducted independently*  Stated in Section 2-3 (Experimental Design and Study Participants). |
|  | 10. | *Describe the statistical analysis*  Stated in the 2-5- Statistical Analysis (aim 2-2). |
| Results | 11. | *State the actual number of raters and subjects/objects that were included and the number of replicate observations that were conducted.*  Stated. Thirty-nine radiologists agreed to participate in the study, and all completed both rounds (no drop-out). For each reader in each round, 160 gist scores corresponding to 160 images were available. |
|  | 12. | *Describe the sample characteristics of raters and subjects (e.g., training, experience)*  Stated (Table 2 and 3). The raw data is available on Kaggle. |
|  | 13. | *Report estimates of reliability and agreement including measures of statistical uncertainty*  Stated. For the mean values the confidence interval and for the median values, min, max, fist and third quartiles were provided. |
| Discussion | 14. | *Discuss the practical relevance of results.*  Stated. In summary, gist signal is not sufficiently reliable. However, our findings suggest that a strong gist of the abnormal, should not be ignored, because when the gist is strong, the probability of a case being abnormal is significantly higher than the probability that it is normal. Conversely, in the absence of a sense of an abnormal gist, readers should keep searching because the absence does not reliably signal a normal case. |
| Auxiliary material | 15. | *Provide detailed results if possible*  In the supplementary materials and the Kaggle all values and raw data are available. |

METHODS
*- Explain how the sample size was chosen. State the determined number of raters, subjects/objects, and replicate observations.
Authors didn’t provide any explanation for sample size for either participants or readers.*

A section is added to the Materials and Methods (Section 2-2) to describe the sample size calculation.  *- Describe the sampling method.
This item was insufficiently reported: authors didn’t describe any method for avoiding selection bias of cases that were very suspicious of malignancy. This should be clearly stated and acknowledged as an important limitation.*

We added further explanation to Section 2-4, where the mammographic images were described. Moreover, in the Discussion, this is mentioned as a limitation of the study.

The images were randomly retrieved from the breast cancer screening archive. Two experienced radiologists confirmed that the images had an acceptable quality in terms of breast positioning and acquisition parameters. “Normal” mammograms came from women who were normal at the time of examination and remained normal in the next round of screening. The experienced radiologists were asked to ensure none of the “Normal” cases contained benign findings or regions suspicious of malignancy.

As stated in Discussion Section, did not include cases with benign findings in our “Normal” category. Moreover, we did not include the normal cases highly suspicious of malignancy. We only focused on two extremes, i.e., normal and abnormal, to study the gist signal. These two categories resulted in low and high gist scores. However, in real clinical practice, radiologists deal with benign findings and highly suspicious normal cases as well. As no benign cases were included here, we cannot exclude that a benign finding might evoke a gist signal as well. This could limit the usefulness of the gist signal for breast cancer detection in real clinical practice.

*- Describe the measurement/rating process (e.g. time interval between repeated measurements, availability of clinical information, blinding).
This item was insufficiently reported: authors didn’t describe whether the order of the images was the same between round 1 and round 2 and among the different readers.*

The time interval between repeated measurements: This is now added to Section 2-3 (Experimental Design and Study Participants) and Section 3-1 (“On average, readers conducted the second round of the readings 7±1 weeks after the first round.”).

Image Order: Images were presented in random order. Now stated in Section 2-3 (Experimental Design and Study Participants). In each session, the images were shuffled, and hence, the order of presentation of images was randomly assigned to each round and each observer.

*- State whether measurements/ratings were conducted independently.*

*Authors didn’t state whether the reader was alone in the room while assessing the images.*

Now stated in Section 2-3 (Experimental Design and Study Participants). They were alone in the room while assessing the images.

*- Describe the statistical analysis.*

*This item was not reported in the methods section. Authors should detail all the statistics that was used and the reason for doing that in the methods section.*

A new section (Section 2-5, Statistical Analysis) is now added to the current revised version of the manuscript to address this comment.

*Additionally, the authors’ criteria for creating a special class of readers, the “Gist Expert” is not adequate. Selecting a population based on the observed performance for making conclusions about their performance in the same dataset their were selected brings a lot of bias. This problem is similar to making conclusions about the diagnostic test accuracy of a test based only on the training dataset, excluding the need for a validation data-set. Authors should try to use baseline criteria to select a group, for example, the third working more hours per week reading mammograms, to evaluate whether reading more mammograms per week improve the readers ability.*

Thank you very much for the comment. In the current study, readers were also asked to complete a comprehensive questionnaire about their workload, practice type, experience levels, age, and gender, and. As shown in Table 2, the Pearson correlation coefficient was used to explore the association between the reader’s performance and the reader characteristics, which were collected as continuous/ordinal variables, including age, number of hours per week currently spent in reading mammograms, number of screening cases you read per week (screening interpretative volume), number of years certified as a BreastScreen reader, percentage of your time is dedicated to reading breast images, percentage of the time dedicated to reading the diagnostic mammograms, duration of fellowship training, number of years since fellowship training, number of years registered as a reader, number of years reading mammograms, number of breast biopsy examinations performed in the last 12 months, how often participate in a Multi-Disciplinary Meeting (MDT) in a month, and how often correlate/review radiology-pathology findings for biopsy cases in a month. For the dichotomous categorical variables (i.e., gender, whether they are radiologists or breast physicians, whether they are a screen reader, whether they are fellowship-trained, whether affiliated with a university or educational institutes, and whether they work full-time), Mann-Whitney U-test was used to investigate if the reader’s performance varied across two categories. However, **none** of the reader characteristics exhibited a significant relationship with the readers’ performance. Therefore, superiority in detecting the gist of the abnormal is an innate reader feature and at present, this capability can be only identified by measuring the performance of the reader on the gist task. Analysis of the overall performance of the readers suggests that, when the gist signal is strong enough, i.e., with the cancer cases, the performance level in the first round of the gist experiment remained almost consistent in the second round. In other words, readers who were “Gist Experts” based on their performance in the first round, were considered “Gist Experts” based on their performance in the second round as well. Therefore, although none of the reader characteristics were predictors of the gist performance, those with the capability of perceiving visible abnormalities based on their first impression, consistently outperformed others in the gist experiment.

*The results section should be greatly reduced. The current presentation is very confusing. I strongly suggest authors to report this section using the following order.*

As per the reviewer’s suggestion, some of the results were moved to the supplementary materials. We also amended the manuscript to ensure that in all three sections of the manuscript (Materials and Methods, Results, and Discussion), the order of presented materials is in concordance with the order of study aims in the Introduction Section.

*RESULTS
- State the actual number of raters and subjects/objects which were included and the number of replicate observations which were conducted.*

*Authors could provide a better description of what was performed and whether there was any problem (for example, whether a reader didn’t return for the round 2).*

None of the readers dropped out of the study. This information is now stated in Section 3-1. We also provided a further description of what was performed in Section 2-3 (Experimental Design and Study Participants).

*- Describe the sample characteristics of raters and subjects (e.g. training, experience).
Authors might present Table 3, but the must add estimates of dispersion (min-max and p25-p75)*

Thank you very much for raising this. In the revised version, Table 3 is updated as suggested.

*- Report estimates of reliability and agreement including measures of statistical uncertainty.
Authors should use a table to present the observed intra- (Round 1 vs Round 2) and inter-rater (Round 1 vs. Round 1) ICC and weighted Cohen’s kappa. Authors interpretation of ICC should also be based on the suggestion from GRRAS:“Values of 0.60, 0.70, or 0.80 are often used as the minimum standards for reliability coefficients, but this may be only sufficient for group-level comparisons or research purposes [12,58,108]. For example, ICC values for a scale measuring pressure ulcer risk should be at least 0.90 or higher when applied in clinical practice [108]. If individual and important decisions are made on the basis of reliability estimates, values should be at least 0.90 [4] or 0.95 [109].”*

We updated Section 3-3 (The intra- and inter-reader reliability of gist responses) and provided a table showing all ICC and kappa values. To report the statistical uncertainty, either confidence interval or min-max and 25th-75th percentiles (in case of Median values) were provided. The ICC values were interpreted as per the reviewer’s suggestion.

*Discussion
The current discussion section brings a lot of speculation regarding the effect of the readers’ first impression of their final evaluation of the mammogram, which should be completely removed as this was not assessed by the study. Authors should focus on: key results, study limitations, and provide a careful interpretation of their results. My only interpretation based on the results of this study is that the intra-, and particularly, inter-observer reliability of the method is very poor and therefore this should not be used in clinical practice.*

Thank you very much for the comment. As suggested, we rewrote the Discussion section. To make it easier to follow, we outlined the key findings in an order similar to the study questions, presented in the Introduction.

As noted by the reviewer, the current study shows that detecting this signal is noisy, with relatively low levels of inter- and intra-reader agreement. Despite the variations across individual readers, a near-perfect agreement in the gist scores for the average reader (the ICC of 0.96 (CI: 0.95-0.97) when comparing the average abnormality ratings from all readers) across the two rounds suggests that the strength of the gist signal is an unvarying innate property of the image, which may or not be perceived by a reader reliably. This opens up the opportunity for radiomic or other approaches to exploit this important, inherent image feature. Moreover, our case-level analysis (aim 4) showed a strong gist of the abnormal should not be ignored, because when the gist is strong, the probability of a case being abnormal is significantly higher than the probability that it is normal. Conversely, in the absence of a sense of an abnormal gist, readers should keep searching because the absence does not reliably signal a normal case. This supports the importance of “discovery scanning”, i.e., coarse screening to detect potential targets before terminating the visual search.
