# Supplementary material for: Reliability of radiologists’ first impression when interpreting a screening mammogram
Source: PLoS One. 2023 Apr 25;18(4):e0284605. doi: 10.1371/journal.pone.0284605 (PMC10128970; doi:10.1371/journal.pone.0284605)
Supplement: Attachment — Submitted filename: Responses_06_02_23.docx [file pone.0284605.s004.docx]

We would like to thank the editor and the reviewer for their valuable comments. The changes, in response to the comment, are highlighted in the manuscript.

Also, all de-identified collected data from radiologists, used in this study will be available through the following Kaggle dataset link:

<https://www.kaggle.com/datasets/zibaga/gist-of-the-abnormal-on-screening-mammograms>

PCB, JMW, SJL, KKE, DX, and ZG was funded by National Health and Medical Research Council (grant number APP1162872). Also, PCB, JMW, SJL, and KKE was funded by National Breast Cancer Foundation (IIRS-18-089). The funders had no role in study design, data collection and analysis, decision to publish, or preparation of the manuscript. We can confirm that we understand the above-mentioned statements will be included as “Funding Statement” in the manuscript.

The authors have declared that no competing interests exist.

**Point by point responses to the comments:**

*Reviewer #3:*

*Authors need to change the abstract and discussion.*

*The observed intra-observer and, particularly, the inter-observer reproducibility (wehghted kappa = 0.3, ICC = 0.3) analyses have clearly shown that the method is not reliable and shouldn't be used in clinical practice. Everything else in the manuscript is only distraction and authors should focus on their main findings.*

Thank you very much for the comment. We rewrote the abstract and most of the Discussion to address this comment. In the revised version of the abstract, we solely explain the results indicating the test-retest reliability of the gist signal to ensure that the main message and finding of the paper are clear to the readers. We also amended the Discussion and mainly focused on discussing the findings concerning the intra- and inter-observer reliability of the signal. We ensured that it is clearly stated in both abstract and discussion that the gist signal has a poor test-retest reliability and hence should not be used in clinical practice.
