# Supplementary material for: Reliability of radiologists’ first impression when interpreting a screening mammogram
Source: PLoS One. 2023 Apr 25;18(4):e0284605. doi: 10.1371/journal.pone.0284605 (PMC10128970; doi:10.1371/journal.pone.0284605)
Supplement: Attachment — Submitted filename: Responses_03_2023.docx [file pone.0284605.s005.docx]

We would like to thank the editor and the reviewer for their valuable comments. The changes, in response to the comment, are highlighted in the manuscript.

Also, all de-identified collected data from radiologists, used in this study will be available through the following Kaggle dataset link:

<https://www.kaggle.com/datasets/zibaga/gist-of-the-abnormal-on-screening-mammograms>

PCB, JMW, SJL, KKE, DX, and ZG was funded by National Health and Medical Research Council (grant number APP1162872). Also, PCB, JMW, SJL, and KKE was funded by National Breast Cancer Foundation (IIRS-18-089). The funders had no role in study design, data collection and analysis, decision to publish, or preparation of the manuscript. We can confirm that we understand the above-mentioned statements will be included as “Funding Statement” in the manuscript.

The authors have declared that no competing interests exist.

**Point by point responses to the comments:**

*Reviewer #3:*

*The last two sentences of the discussion section are speculative and should be removed:*

*"Despite the variations across individual readers, a near-perfect agreement in the gist scores for the average reader across the two experiments suggests that the strength of the gist signal is an unvarying innate property of the image, which may or not be perceived by a reader reliably. This opens up the opportunity for radiomic [36] or other approaches to exploit this important, inherent image feature.".*

Thank you very much for the comment. As suggested, the last two sentences were omitted in the revised manuscript.
